# Serum hsa_circ_0079480 is a novel prognostic marker for acute myeloid leukemia

**DOI:** 10.1002/jcla.24337

**Published:** 2022-03-17

**Authors:** Liang Guo, Ru Kou, Yanping Song, Guang Li, Xueyou Jia, Zhenzhen Li, Yunjie Zhang

**Affiliations:** ^1^ Institute of Hematopathy Xi'an Central Hospital Xi'an China; ^2^ Department of Clinical Laboratory Xi'an Central Hospital Xi'an China

**Keywords:** acute myeloid leukemia, hsa_circ_0079480, prognostic marker

## Abstract

**Background:**

The dysregulated expression of serum circular RNAs (circRNAs) has previously been linked to the prognosis of acute myeloid leukemia (AML) patients, but the clinical and prognostic relevance of serum hsa_circ_0079480 levels in this oncogenic setting have yet to be established. Herein, we assessed the putative prognostic relevance of circulating hsa_circ_0079480 levels in AML patient serum.

**Methods:**

Serum was prepared from blood samples collected from 236 AML patients and 160 healthy controls, with hsa_circ_0079480 levels therein being quantified by quantitative real‐time reverse transcription‐polymerase chain reaction (qRT‐PCR) after which the clinical value of these levels was assessed.

**Results:**

Acute myeloid leukemia patients were found to exhibit significant hsa_circ_0079480 upregulation in their serum as compared to serum from healthy controls, with such upregulation being most profound in individuals with M4/M5 type disease and to be more common in patients with poor cytogenic risk or high white blood cell counts. Receiver operating characteristic (ROC) curves demonstrated that serum hsa_circ_0079480 levels were able to effectively differentiate between patients with AML and healthy controls. Moreover, the upregulation of serum hsa_circ_0079480 was found to be closely related to clinicopathological findings and to be an independent predictor of reduced overall and relapse‐free survival among individuals diagnosed with AML. Furthermore, serum hsa_circ_0079480 levels were markedly decreased after treatment in this patient population, with these levels being lower in patients that achieved complete remission as compared to those patients that did not.

**Conclusion:**

Levels of hsa_circ_0079480 in patient serum may offer value as a prognostic biomarker in AML.

## 1 INTRODUCTION

Acute myeloid leukemia (AML) is a form of aggressive hematopoietic cancer that results from clonal myeloid precursor cell proliferation,^1^, ^2^ ultimately resulting in leukemic blast accumulation within the blood, bone marrow, and tissues together with significant reductions in levels of non‐malignant blood cells.^3^ Cytogenic factors can be used to stratify AML patients into the poor, intermediate, and favorable cytogenic risk categories.^4^ Despite important advances in the treatment of AML, the 5‐year overall survival (OS) of affected patients is relatively low (11–55%).^5^, ^6^, ^7^, ^8^ There is thus a clear need to identify prognostic biomarkers capable of stratifying AML patients based on their risk level to guide more appropriate treatment and clinical management efforts.^9^, ^10^

Circular RNAs (circRNAs) exhibit a covalently closed loop‐like morphology and lack coding potential, 5′ caps, and 3′ polyadenylated tails.^11^, ^12^ These circRNAs can regulate the immune response, oncogenic progression, and chemoresistance among other processes.^13^, ^14^ Mechanistically, circRNAs can bind to specific microRNAs (miRNAs) in a sequence‐specific manner, thereby serving as competing endogenous RNAs (ceRNAs) to prevent miRNA‐mediated repression of mRNA expression. For example, in AML, hsa_circ_0001947 can inhibit disease progression by regulating the hsa‐miR‐329‐5p/CREBRF axis,^15^ while circ_0006404 enhances the progression of prostate cancer progression via the miR‐1299/CFL2 axis,^16^ and hsa_circ_0001073 can suppress the progression of lung cancer via modulating miR‐626/LIFR axis signaling.^17^

In previous studies, hsa_circ_0079480, which is encoded on chr7:16298014‐16317851, has been identified as an oncogenic driver of AML capable of promoting tumor progression through the miR‐654‐3p/HDGF regulatory axis.^18^ The clinical importance of serum hsa_circ_0079480 in AML patients, however, has yet to be established, and this study was therefore designed to examine the prognostic value of hsa_circ_0079480 levels in the serum of individuals with AML.

## 2 MATERIALS AND METHODS

### 2.1 Sample collection

In total, 160 healthy controls and 236 newly diagnosed AML patients were included in the present analysis. AML patients were diagnosed and classified as per French‐America‐British (FAB) and World Health Organization criteria, with corresponding characteristics being compiled in Table 1. Three cytogenic risk level‐based subgroups were established (poor, intermediated, and favorable). For further details regarding AML patient cytogenic information, see Table 2. Complete remission (CR) was defined by the detection of <5% blast cells in the bone marrow and normal peripheral blood counts 4 weeks following the initiation of induction therapy, with no residual signs of extramedullary disease. Overall survival (OS) was defined by the amount of time between patient diagnosis and all‐cause mortality or most recent follow‐up, while relapse‐free survival (RFS) was the time between CR and disease recurrence or most recent follow‐up. Similar chemotherapeutic regimens were used to treat all AML patients in the present study cohort, with induction chemotherapy being administered based on clinical status using protocols that were designed with references to the NCCN Clinical Practice Guidelines for AML. Serum hsa_circ_0079480 levels were assessed in both pre‐ and post‐treatment samples from these AML patients. The Ethics Committee of Xi'an Central Hospital approved this study, with all patients having provided written informed consent.

**TABLE 1:** Relationships between serum hsa_circ_0079480 and AML patient clinicopathological findings

Characteristics	Number	hsa_circ_0079480		*p*
		Low expression	High expression	
Age (years)				
<60	159	76	83	0.323
≥60	77	42	35	
Gender				
Male	126	66	60	0.624
Female	110	52	58	
BM blasts (%)				
<50	109	49	60	0.119
≥50	127	69	58	
WBC counts (×10^9^/L)				
<10	85	58	27	0.035
≥10	151	60	91	
FAB subtype				
M0	21	12	9	0.215
M1/M2	137	72	65	
M4/M5	78	34	44	
Platelet counts (× 10^9^/L)				
<50	121	56	65	0.084
≥50	115	62	53	
Cytogenetics				
Favorable	92	59	33	0.009
Intermediate	112	50	62	
Poor	32	9	23	
Complete remission				
Yes	107	55	52	0.117
No	129	63	66	

Abbreviations: AML, acute myeloid leukemia; FAB, French‐America‐British; WBC, white blood cell.

**TABLE 2:** Multivariate analysis of independent predictors of AML patient prognosis

Characteristics	Risk ratio	95% CI	*p*
OS (all AML, N = 236)			
WBC counts (×10^9^/L)	3.01	1.53–3.75	0.032
Cytogenetics	3.21	1.61–4.84	0.017
Serum hsa_circ_0079480	3.19	1.59–4.62	0.009
RFS (all AML, N = 236)			
WBC counts (×10^9^/L)	3.15	1.61–4.12	0.022
Cytogenetics	3.62	1.74–5.02	0.012
Serum hsa_circ_0079480	3.48	2.01–5.42	0.002

Abbreviations: AML, acute myeloid leukemia; OS, overall survival; RFS, relapse‐free survival; WBC, white blood cell.

Serum samples were obtained by harvesting fasting peripheral venous blood from 160 healthy controls and 236 patients with AML. After collection, samples were initially centrifuged for 10 min at 2500 *g*, followed by additional centrifugation for 10 min at 16,000 *g*. Serum was then stored at −80°C pending subsequent analysis.

### 2.2 Quantitative real‐time reverse transcription‐polymerase chain reaction

A miRNeasy Serum/Plasma kit (Qiagen) was utilized based on provided directions to extract total RNA, the concentration of which was then quantified using a NanoDrop 2000 spectrophotometer (Thermo Fisher Scientific). A PrimeScript RT Reagent Kit (TaKaRa) was then used to prepare cDNA, with quantitative real‐time reverse transcription‐polymerase chain reaction (qRT‐PCR) analyses being performed with an ABI 7500 instrument (ABI) using a SYBR Premix EX Taq Kit (TaKaRa). The 2^−ΔΔCt^ method was utilized to examine relative gene expression. Primers used for these analyses included: hsa_circ_0079480‐F, 5′‐CAACCTCTGATTTTCAAGAAACC‐3′ and hsa_circ_0079480‐R, 5′‐TTCACTTTTCAGCACTTCTTCAA‐3′.

### 2.3 Statistical analysis

Levels of circulating hsa_circ_0079480 were compared between groups via Student's t tests and one‐way ANOVAs, while relationships between this circRNA and clinicopathological variables were assessed via chi‐squared tests. The ability of serum has_circ_0079480 to differentiate between patients with and without AML was assessed based on the area under the receiver operating characteristic (ROC) curve (AUC). Survival outcomes were assessed using Kaplan–Meier curves and the log‐rank test. Independent predictors of patient outcomes were identified through multivariate Cox proportional hazards regression analyses. GraphPad Prism 7 (GraphPad Software) and SPSS 16.0 (SPSS Inc.) were used for all analyses, with a *p* < 0.05 as the significance threshold.

## 3 RESULTS

### 3.1 AML patients exhibit increased serum hsa_circ_0079480 expression

**FIGURE 1:**
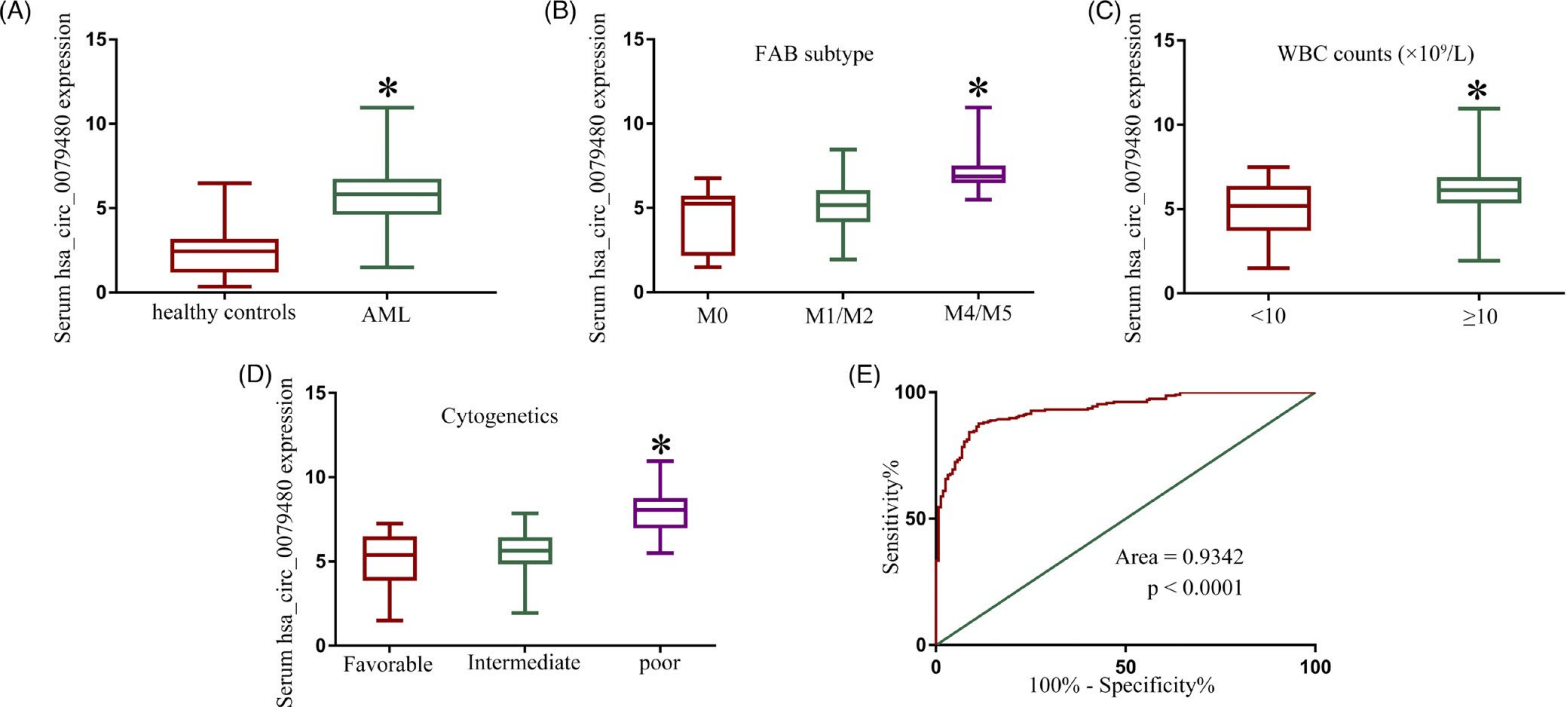
Acute myeloid leukemia (AML) patients exhibit increased serum expression of hsa_circ_0079480. (A) Relative to healthy controls, AML patients exhibited significantly higher serum hsa_circ_0079480 expression. (B) AML patients with M4/M5 disease exhibited higher serum hsa_circ_0079480 levels as compared to those in individuals with M1/M2 disease. (C) Levels of serum hsa_circ_0079480 were significantly elevated in patients with higher WBC counts. (D) Levels of hsa_circ_0079480 were increased in the serum of patients with AML exhibiting poor cytogenic risk. (E) hsa_circ_0079480 levels in AML patient serum were able to effectively discriminate between AML patients and controls. *p < 0.05

We began by using a qRT‐PCR approach to compare serum hsa_circ_0079480 levels in 236 AML patients and 160 healthy controls, revealing the significant upregulation of this circRNA in these cancer patients relative to corresponding controls (Figure 1A). Lower hsa_circ_0079480 levels were more commonly observed among AML patients with M1/M2 disease relative to patients with M4/M5 disease (Figure 1B). Additionally, levels of this serum circRNA were lower in AML patients with a white blood cell (WBC) count <10 × 10^9^/L relative to levels in patients with a WBC count above this threshold (Figure 1C). Circulating levels of hsa_circ_0079480 were also significantly elevated in the poor cytogenetic risk subgroup relative to the intermediate and favorable cytogenic risk subgroups, with these levels similarly being higher in the intermediate cytogenic risk group as compared to the favorable cytogenetic risk group (Figure 1D). ROC curve analyses revealed serum hsa_circ_0079480 levels to effectively differentiate between AML patients and healthy controls at an AUC of 0.9342 (Figure 1E).

### 3.2 Serum hsa_circ_0079480 levels are correlated with AML patient clinicopathological findings

Next, AML patients in this study cohort were stratified into subgroups with low and high levels of serum hsa_circ_0079480 (n = 118 each). Elevated levels of this circRNA were significantly correlated with WBC counts and cytogenetics, but were unrelated to patient age, CR status, platelet count, FAB subtype, or bone marrow blasts.

### 3.3 Elevated serum hsa_circ_0079480 levels are linked to worse AML patient survival outcomes

**FIGURE 2:**
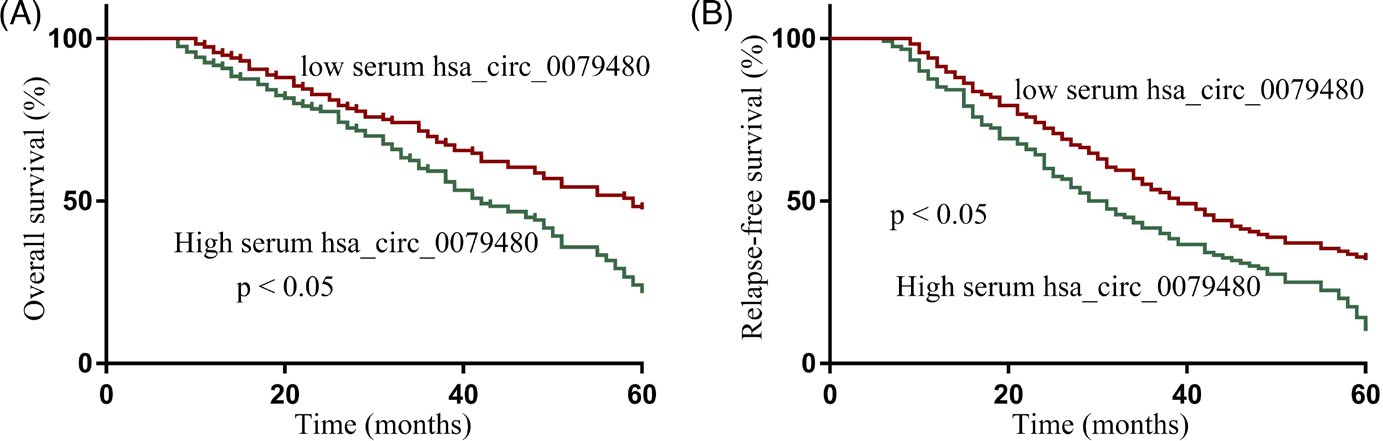
Upregulation of hsa_circ_0079480 in acute myeloid leukemia (AML) patient serum is related to worse survival outcomes. AML patients with higher levels of serum hsa_circ_0079480 exhibited worse overall survival (OS) (A) and relapse‐free survival (RFS) (B) as compared to patients with lower serum levels of hsa_circ_0079480

Through Kaplan–Meier analyses and log‐rank tests, high serum hsa_circ_0079480 levels were found to be significantly associated with reduced AML patient OS (Figure 2A) and RFS (Figure 2B) as compared to patients exhibiting lower circulating levels of this circRNA.

### 3.4 Serum hsa_circ_0079480 levels are independently associated with AML patient prognosis

In a multivariate analysis, WBCs (RR = 3.01; 95% CI = 1.53–3.75, *p* = 0.032), cytogenetics (RR = 3.21; 95% CI = 1.61–4.84, *p* = 0.017), and serum hsa_circ_0079480 levels (RR = 3.19; 95% CI = 1.59–4.62, *p* = 0.009) were all found to be significantly associated with AML patient OS. Additionally, WBCs (RR = 3.15; 95% CI = 1.61–4.12, *p* = 0.022), cytogenetics (RR = 3.62; 95% CI = 1.74–5.02, *p* = 0.012), and serum hsa_circ_0079480 levels (RR = 3.48; 95% CI = 2.01–5.42, *p* = 0.002) were independently associated with AML patient RFS.

### 3.5 AML patient treatment is associated with altered serum hsa_circ_0079480 levels

**FIGURE 3:**
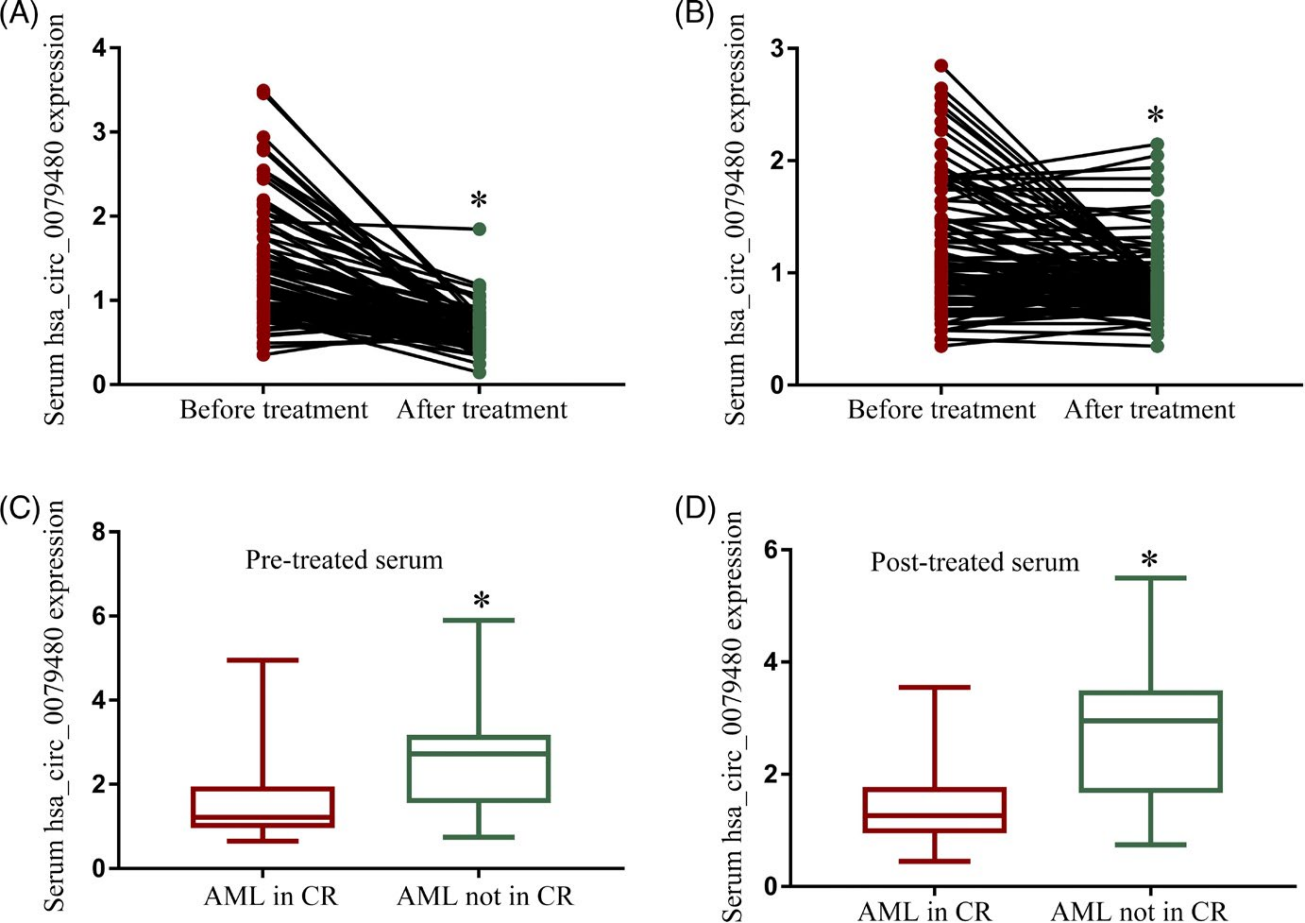
Post‐treatment changes in hsa_circ_0079480 levels in the serum of acute myeloid leukemia (AML) patients. (A‐B) In AML patients that achieved complete remission (CR) (A) or did not achieve CR (B), marked reductions in serum hsa_circ_0079480 were evident in post‐treatment samples as compared to pre‐treatment samples. (C‐D) Serum hsa_circ_0079480 expression was significantly reduced in both pre‐ and post‐treatment samples from AML patients that did achieve CR relative to those from patients that did not. *p < 0.05

On Day 28 after the first chemotherapy cycle, blood samples collected from patients with AML were analyzed to evaluate changes in serum hsa_circ_0079480 levels that may be correlated with treatment outcomes. Out of 236 AML patients included in the present study, 112 and 124 did and did not achieve CR. Serum hsa_circ_0079480 levels fell in both of these patient subgroups after the treatment (Figure 3A–B), but those patients that did achieve CR exhibited significantly reduced serum hsa_circ_0079480 levels both before and after the treatment when compared to patients that did not achieve CR (Figure 3C–D). As such, the serum levels of this circRNA may offer value as a tool for monitoring AML patient therapeutic responses.

## 4 DISCUSSION

Owing to their evolutionary conservation, stability, and looped structure, circRNAs have been a focus of growing research interest.^19^, ^20^, ^21^ There is growing evidence that circRNA dysregulation in AML is functionally important and can shape the onset and progression of this disease. For example, circ‐PTK2 can inhibit the apoptotic death of AML cells while promoting their proliferative activity by regulating the miR‐330‐5p/FOXM1 axis.^22^ The knockdown of circ_0058058 can suppress the progression of AML by modulating the sequestration of miR‐4319 and thereby regulating the expression of EIF5A2.^23^ AML samples exhibit the upregulation of circ_POLA2, which can inhibit mature miR‐34a production and thereby promote cellular proliferation.^24^ Moreover, circRNAs can offer value as diagnostic biomarkers in this oncogenic context, as in the case of circ_0002232, which offers value as a biomarker of AML that has been posited to function through a circ_0002232/miR‐92a‐3p/PTEN ceRNA regulatory pathway.^25^ Other circRNA profiling and bioinformatics studies have also identified hsa_circ_0004277 as a promising AML‐related biomarker,^26^ and the annexin A2 circRNA has also been highlighted as a promising biomarker and therapeutic in this cancer.^27^

Herein, we determined that AML patients exhibited significant increases in serum hsa_circ_0079480 levels as compared to healthy controls, with ROC analyses confirming the good performance of serum hsa_circ_0079480 when used to discriminate between AML patients and healthy individuals. One potential explanation for the observed dysregulation of this circRNA may be that tumor cells synthesize and/or secrete higher levels of hsa_circ_0079480 into systemic circulation. Higher levels of hsa_circ_0079480 in patient serum were also associated with more aggressive clinicopathological findings and worse patient survival outcomes, suggesting that such patients may benefit from more assertive treatment. Multivariate analyses further revealed elevated serum levels of this circRNA to be an independent predictor of AML patient OS and RFS, indicating that it may offer value as a prognostic biomarker in this cancer type. Notably, levels of hsa_circ_0079480 were significantly lower among AML patients undergoing treatment, particularly in individuals that achieved CR, suggesting that serum hsa_circ_0079480 can be assessed as a sensitive biomarker associated with patient therapeutic responsiveness. Overall, increases in serum hsa_circ_0079480 offer promise as a biomarker associated with AML onset and development, in line with prior evidence for the oncogenic activity of hsa_circ_0079480 in AML.^18^

One potential limitation of this analysis is the fact that other cancers and diseases may result in the dysregulation of serum hsa_circ_0079480, suggesting that this circRNA may need to be combined with other clinical details and relevant biomarkers to accurately predict AML patient outcomes. Additionally, further research is required to clarify the mechanistic role of hsa_circ_0079480 as a regulator of AML progression.

## 5 CONCLUSION

In summary, we herein found that AML patients exhibit marked increases in serum hsa_circ_0079480 levels, with such upregulation being significantly associated with aggressive disease progression and poorer patient prognosis. As such, serum hsa_circ_0079480 may offer value as a prognostic biomarker in patients with AML.

## CONFLICT OF INTEREST

The authors declared no conflicts of interest.

## Data Availability

Due to the nature of this research, participants of this study did not agree for their data to be shared publicly, so supporting data are not available.
